# Impact of Meditation–Based Lifestyle Modification on HRV in Outpatients With Mild to Moderate Depression: An Exploratory Study

**DOI:** 10.3389/fpsyt.2022.808442

**Published:** 2022-06-09

**Authors:** Holger C. Bringmann, Martin Bogdanski, Georg Seifert, Andreas Voss

**Affiliations:** ^1^Institute of Social Medicine, Epidemiology, and Health Economics, Charité – Universitätsmedizin Berlin, Corporate Member of Freie Universität Berlin and Humboldt-Universität zu Berlin, Berlin, Germany; ^2^Department of Psychiatry, Psychosomatics, and Psychotherapy, Spremberg Hospital, Spremberg, Germany; ^3^Department of Pediatric Oncology and Hematology, Charité – Universitätsmedizin Berlin, Corporate Member of Freie Universität Berlin and Humboldt-Universität zu Berlin, Berlin, Germany; ^4^Institute of Biomedical Engineering and Informatics (BMTI), Technische Universität Ilmenau, Ilmenau, Germany

**Keywords:** yoga, meditation, ethics, mantra, Lifestyle Modification, HRV, depression, psychiatry

## Abstract

**Background:**

The scientific evaluation of mind-body-interventions (MBI), including yoga and meditation, has increased significantly in recent decades. However, evidence of MBI's efficacy on biological parameters is still insufficient.

**Objectives:**

In this study, we used HRV analysis to evaluate a novel MBI as a treatment of outpatients with mild to moderate depressive disorder. The Meditation-Based Lifestyle Modification (MBLM) program incorporates all major elements of classical yoga, including ethical principles of yoga philosophy, breathing exercises, postural yoga, and meditation.

**Methods:**

In this exploratory randomized controlled trial, we compared the changes in HRV indices of a MBLM group (*N* = 22) and a minimal treatment group (MINIMAL, drugs only, *N* = 17) with those of a multimodal treatment-as-usual group (TAU, according to best clinical practice, *N* = 22). Electrocardiogram (ECG) recordings were derived from a Holter monitoring device, and HRV indices have been extracted from nearly stationary 20-min periods.

**Results:**

Short-term HRV analysis revealed statistically significant differences in the pre-to-post changes between MBLM and TAU. In particular, the vagal tone mediating RMSSD and the Rényi entropy of symbolic dynamics indicated HRV gains in MBLM participants compared with TAU. Almost no alterations were observed in the MINIMAL group.

**Conclusions:**

Our results suggest a benefit in selected HRV parameters for outpatients with mild to moderate depression participating in the MBLM program. For further investigations, we propose analysis of complete 24-h HRV recordings and additional continuous pulse wave or blood pressure analysis to assess long-term modulations and cardiovascular effects.

## Introduction

As an easy to obtain and non–invasive method to determine the regulative capacities of the autonomous nervous system (ANS), the assessment of heart rate variability (HRV) has been established to investigate different types of stress-related cardiovascular (dys-) functions as well as mental disorders. Particularly in the last decade, the field of studies examining the effects of various mind-body-interventions (MBI) on HRV has grown. These involved studies on yoga (1–3) as well as mindfulness and other meditative interventions (4–8).

HRV can be interpreted as a marker of sympatho-vagal balance and/ or imbalance of the ANS. Typically, a higher HRV at rest stands for better health and adaptability of the body to various types of stressors. In contrast, it can be impaired by disease or chronic stress, and acts as a predictive marker for the risk of further diseases (9–12). These include primarily somatic conditions like cardiovascular- or coronary heart diseases (13–20), and inflammatory processes related to the immune system (17, 21–23). But also, psychological disorders like burnout, anxiety, or depression (24–26) are correlated with a compromised HRV. Vice versa, an improvement in HRV represents an indicator of an improvement in physical and / or mental health.

Regarding the influence of yoga on HRV, some positive effects were found in systematic reviews and meta-analyses, but with the limitation of an overall insufficient study quality and partly contradictory results (1, 3). A recent meta-analysis on mindfulness and meditation concluded that there is currently insufficient evidence to indicate that MBIs lead to improvements in HRV over control conditions (8). Still, MBIs are increasingly used in the treatment of depression and HRV has been suggested as an indicator of success (27) or predictor of the outcome in depression treatment (28). HRV has also been investigated as a biofeedback treatment to alleviate symptoms of patients with Major Depression Disorder (29).

Beyond the use of HRV as a clinical biomarker of depression, HRV (among others) has been proposed as an important index of central-peripheral integration and homeostasis, elucidating the potentially underlying mechanisms of MBIs in general. Taylor, Goehler (30) propose an integrative framework for psychophysiological research based on the interactions of neurocognitive top-down and neurophysiological bottom-up processes that contribute to both mental and physical health in MBIs. In more specific frameworks of self-regulation, these principles of interacting top-down and bottom-up processes were applied to classical yoga, which comprises ethics, postures, breath regulation and meditation (31).

The Meditation-Based Lifestyle Modification (MBLM) program (32) is a second-generation MBI, that is particularly representative of classical yoga. Unlike most MBIs, the program includes not only postural yoga, breathing exercises and/or meditation, but particularly the ethical principles of yoga philosophy. In terms of the aforementioned frameworks, the program equally serves neurocognitive and neurophysiological processes for eudaimonic wellbeing. The program has been shown to be feasible and effective for depressive outpatients (33, 34). In healthy individuals participating in the MBLM program, incremental and differential effects of practicing various components of the program could be seen, supporting the relevance of multilayered processes as described above (35).

In the present, exploratory analysis, we evaluate the effects of MBLM on alterations of HRV indices in depressive outpatients. The aim was to assess whether the MBLM program, as an integrated approach to the treatment of depression, results in an improvement of autonomic regulation compared to two reference groups (treatment as usual and minimal treatment).

## Materials and Methods

### Ethical Approval and Informed Consent

All procedures performed in the study involving human participants were approved by the Ethics Review Board of Chemnitz University of Technology (V-276-15-PS-MBLM-D- 14062018). Informed consent was obtained from all subjects participating in the study before they were randomly assigned to treatment groups. They were informed about their assignment after randomization.

### Study Design

This study was designed as an exploratory three-arm, randomized controlled trial (ClinicalTrials.gov Identifier: NCT03652220) to investigate measurable effects on participant's HRV parameters with respect to 8 weeks of different treatment conditions for patients with mild to moderate depression. It was conducted at the Clinic for Psychiatry, Psychosomatics, and Psychotherapy, Zschadraß, Germany from July 2018 through June 2020. Prior to the interventions, the participants were randomly assigned to one of the three groups: intervention group (MBLM), minimal treatment group (MINIMAL) or treatment as usual (TAU). All participants conducted mobile 24-h electrocardiogram (ECG) measurements prior to the interventions and after 8 weeks.

### Participants

**Figure 1:**
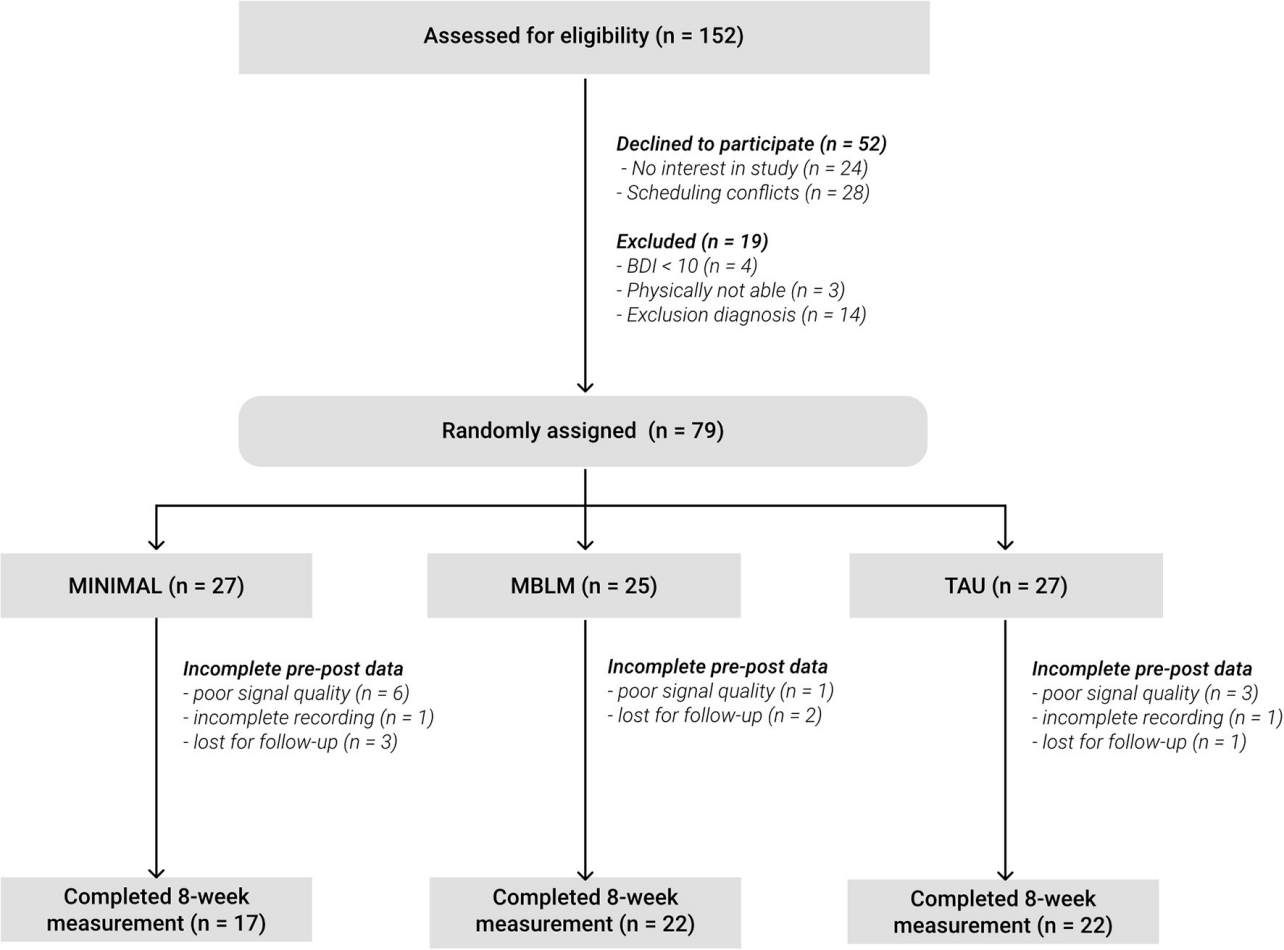
Participant flow.

The recruitment of non-stationary patients was realized *via* flyers posted in the waiting room of the outpatient department at the psychiatric clinic and *via* their attending psychiatrist. The eligibility criteria included outpatients who had been diagnosed with mild or moderate depression according to ICD-10 criteria, who achieved a total score of at least 10 points in the Beck Depression Inventory (BDI-II) (36) and who were at least 18 years old. An additional requirement for participating was being physically able to sit still for 20 min and to do gentle yoga exercises. Patients with self-reported addictive disorders, psychotic symptoms, acute suicidality, obsessive-compulsive disorder, cerebral organic diseases, or disrupted circadian control of heart rate were excluded. The randomization into the three groups was implemented by a software-based minimization algorithm (37, 38) to control for equal group sizes and homogeneous subpopulations in terms of age, gender, BDI-II scores, adverse childhood events and recurrence of depression. Of the *N* = 79 participants, data of 18 patients had to be discarded due to drop-out or insufficient signal quality of the recordings (for details, see Figure 1). Some demographic and additional information is given in Table 1.

**Table 1:** Participant information.

		**Groups**		
**Label**	**Levels**	**MBLM**	**MINIMAL**	**TAU**
Age	Mean (SD)	49.1 (11.1)	51.0 (12.7)	45.0 (11.1)
Gender	Female	21 (77.8)	21 (77.8)	23 (85.2)
	Male	6 (22.2)	6 (22.2)	4 (14.8)
Cigarettes per day	Mean (SD)	2.7 (6.0)	6.0 (7.7)	4.2 (6.8)
Alcoholic bev. per week	Mean (SD)	1.1 (1.6)	1.8 (3.3)	1.2 (1.8)
Depression type	First Episode	10 (37.0)	11 (40.7)	10 (37.0)
	Recurrent episodes	17 (63.0)	16 (59.3)	17 (63.0)
Years since first episode	Mean (SD)	6.4 (7.9)	8.2 (8.1)	10.1 (8.5)
ACE ≥ 4	No	19 (73.1)	19 (76.0)	17 (73.9)
	Yes	7 (26.9)	6 (24.0)	6 (26.1)
Months of psychotherapy	Mean (SD)	25.1 (27.0)	24.1 (29.1)	30.4 (29.7)

*ACE, Adverse Childhood Events; MBLM, Meditation Based Lifestyle Modification; MINIMAL, Drug continuation therapy; TAU, Treatment as usual*.

### Treatment Conditions

During the eight–week intervention all participants continued their antidepressant medication, if any. In addition, participants were subjected to different treatment protocols and conditions depending on their respective group, which are described below.

#### Meditation Based Lifestyle Modification (MBLM)

MBLM is a novel mind-body program based on the eight–fold path of classical yoga (32, 34). Participants took part in an eight–week intervention including three domains of practice: ethical living, healthy lifestyle, and mantra meditation. Each of the eight sequential, weekly group sessions of 180 min and additional 45 min of daily home practice involved these three domains. As part of the ethical living domain, the participants learned major aspects of yoga practice regarding virtue-based ethics, e.g., non–violence, truthfulness etc. The second domain, healthy lifestyle, included breathing techniques and gentle yoga exercises as well as some basic advice on healthy lifestyle according to Traditional Indian Medicine (e.g., biorhythm and nutrition). In the third domain, silent mantra meditation was practiced in the group, where the participants recited a mantra inwardly, which was chosen from a list as part of an introductory session prior to the MBLM program. An overview of the MBLM concept is given in the (Supplementary Figure S1).

#### Drug Continuation Therapy (MINIMAL)

Patients in the MINIMAL group continued existing antidepressant medication and received at least one appointment with their attending psychiatrist. Participants in the MINIMAL group did not receive any additional therapies.

#### Treatment as Usual (TAU)

In the TAU group, the participants underwent an individualized multimodal therapy approach for patients with mild to moderate depressive disorders. According to best clinical practice based on the national guidelines for the treatment of unipolar depression [S3-Leitlinie; (39)], this treatment included an individually tailored psychiatric and psychotherapeutic treatment, as well as accompanying therapies conveyed by specially trained nurses, movement therapists, occupational therapists, and social workers. Mind-body therapies like yoga, mindfulness-based stress reduction (MBSR), or mantra meditation, were excluded from the participant's treatment plan during the study because of their similarity to MBLM.

### Data Acquisition

To analyze the effects on all participant's HRV and to compare the outcome of the three different groups, 24-h continuous ECG recordings were performed prior and after end of the eight–week intervention. This was conducted *via* a mobile ECG device of type “eMotion Faros 180”, which was applied by trained staff at the outpatient department and then carried by the participant during daily routine for 24 h. Recordings were saved as European Data Format (EDF) files containing a single-channel ECG at 1 kHz, a three-axis accelerometer at 100 Hz as well as a HRV channel with beat-to-beat intervals (RR) at 4 Hz.

### Pre-processing

Before computation and analysis of HRV indices, some pre-processing procedures using MathWorks MATLAB (Version R2021a) were performed to ensure suitable signal states.

In a first step, the complete 24-h ECG recording was trimmed to a daytime window and the corresponding RR time series detected from the ECG signal was stored to a separate file. For peak detection in this first stage, a wide time window of several h (during the daytime of recording) was used. More specifically, we generally declared 0–5 am as night and took the part before or after night as day phase, depending on which part was longer on the specific 24-h recording. We also verified sleep and night phases *via* information derived from the device's built-in accelerometer and manually adjusted timings if necessary. That means, we have reviewed and evaluated the synchronized movement signal for individual sleep phases. This also included a more detailed look at the morning and evening datetimes of each subject as well as potential sleeping during daytime. Subjects' very early or late individual daytimes or sleep were not considered when selecting the final time window for analysis.

In a second step, we have applied a R-peak detection *via* a wavelet-based approach with additional post-correction for exact peak values. Initially, a suitable wavelet type replicating the shape of an ECG QRS complex was used to find the positions of all QRS within the given ECG signal (40). After that, the exact R-peak location nearby every found QRS position was determined. The RR interval series could then be derived from the obtained R-peak locations. The resulting RR series were then processed by adaptive filtering to correct the signal for ectopic or missed beats and other artifacts to provide normal-to-normal beat interval (NN) series. In the next step of preprocessing, the whole daytime NN series were trimmed to 20 min of nearly stationary signals reflecting comparable and stable variability at a resting condition for further short-term HRV analysis (9, 41). A combination of an automatic estimation algorithm based on mathematical equations and manual evaluation by visual inspection was used to find a suitable 20-min window of (trend-) stationarity with smooth and regular rhythm. At the same time, we visually examined the possible target windows for typical and representative NN values for a resting condition avoiding sleep, exercise, or stress. This has been done with respect to mean heart rate, avoiding minimum or maximum periods as well as strong changes in dynamics.

The variations within the obtained series of beat-to-beat intervals are referred as HRV and can be expressed in a variety of parameters in time- and frequency domains as well as symbolic and other non-linear dynamics.

During the pre-processing, a total of 10 cases have been discarded due insufficient signal. The artifacts and distortions were presumably caused mainly by movement of the participants or loosened fit of the recording device. This included cases with changes >5% of the total daytime window performed by the adaptive filter. However, some cases could be retained if most of the changes were not made within the targeted 20-min window and therefore the final relative proportion was <5%.

In this short-term HRV analysis we decided to focus on NN intervals from daytime. This offered us the opportunity to create a more comparable situation. During night phases, REM stages are more difficult to detect and eliminate. In addition, there were some cases of sleep disorders or at least restless sleep, which would have made it difficult to obtain a situation as homogeneous as possible for the evaluation. This would then have led to further case exclusions and thus reduced the statistical power.

### Feature Extraction

The following seven HRV features where calculated based on the obtained 20 min NN series. The feature extraction process included methods from time- and frequency domains as well as symbolic and non-linear dynamics. These parameters represent information describing the characteristics of the given NN time series and therefore serve as indicators of the participants' autonomic regulation at record time. As proposed by the Task Force of the European Society of Cardiology and the North American Society of Pacing and Electrophysiology, the following standard HRV parameters were computed (9):

- meanNN—mean value of all NN-intervals in [ms]
- sdNN—standard deviation (sd) of all NN-intervals in [ms]
- sdaNN5—standard deviation of the averages of NN-intervals in all 5-min segments in [ms]
- RMSSD—square root of the mean squared differences of successive NN-intervals in [ms]
- pNN50—proportion derived by dividing the number of interval differences of successive NN-intervals >50 ms by the total number of NN-intervals
- LF/HF—ratio of low frequency (0.04–0.15 Hz) and high frequency (0.15–0.4 Hz) power spectra estimates

Additionally, we calculated word probabilities as markers of symbolic dynamics of the HRV. This is an extension into another field of biosignal analysis and could contribute to a wider and more diverse HRV feature set across different domains. Symbolic dynamics as a non-linear approach might provide results not addressed by linear (time and frequency) methods. In the symbolic dynamics in HRV we define a set of characters from an alphabet of A_SymDyn_ = [0–3] which form symbols and result in specific words. These words of three consecutively occurring symbols represent relations of each variation within the given NN time series: indicating rising, falling, or constant successive values. The derived word probabilities for all possible patterns describe the variation sequence. This is expressed as pW000, pW001 … pW333 with symbols “1” and “3” as measure for increased, and the symbols “0” and “2” for decreased variability. As a quantification of the NN-series' complexity, the Rényi entropy can be stated as a summarizing index of the occurring word probabilities. In this case, the parameter SymDynRenyi4 (with α = 4) indicates the randomness and thus the diversity of distribution of the patterns in the present NN series as a measure of the non-linear dynamics of HRV (20, 42).

### Statistical Analysis

To test the difference between the study groups regarding HRV outcomes, we performed a one-way multivariate analysis of variances (MANOVA) with group as between-subject factor (MBLM, TAU or MINIMAL) and the pre-post differences of the HRV parameters as dependent variables (meanNN, sdNN, sdaNN5, RMSSD, pNN50, LF/HF, and SymDynRenyi4). The statistical comparison was implemented in IBM SPSS Statistics (Version 27) *via* the GLM function (General Linear Model) at an alpha level of 0.05.

In addition to the multivariate results *post-hoc* tests based on a model fitting and observed data, respectively, were calculated with contrasts and pairwise comparisons in terms of estimated marginal means (EMMEANS) and multiple comparison testing (MCT). Differences between factor levels using the EMMEANS procedure were examined based on adjusted Sidak tests as a slightly less conservative method than the Bonferroni correction, but with better protection against Type-I errors than the Least Significant Difference (LSD). For *post-hoc* MCT, Dunnett-T adjustment was implemented for Type-I error protection, which combines a moderately conservative protection against Type-I errors with good statistical power. In all *post-hoc* tests, we compared MBLM and MINIMAL against TAU, as the latter was considered the standard treatment according to best clinical practice.

Finally, we calculated the effect size Cohen's *d* as a complementary measure of group differences. According to literature, we considered effect sizes as small (*d* ≥ 0.20), medium (*d* ≥ 0.50) and large (*d* ≥ 0.80) (43).

## Results

The results of the one-way MANOVA showed no statistically significant group effect on the combined dependent variables, *F*(14, 104) = 1.255, *p* = 0.249, partial η^2^ = 0.144, Wilk's Λ = 0.732. That means, that there were no statistically significant differences across all three groups regarding all dependent variables in the multivariate model.

However, the pairwise comparisons based on EMMEANS revealed two (out of seven) statistically significant differences between MBLM and TAU. This applies to RMSSD, *p* = 0.015 (*M*_Diff_ = 4.145, 95%–CI[0.646, 7.644]), and SymDynRenyi4, *p* = 0.021 (*M*_Diff_ = 0.24, 95%–CI[0.029, 0.452]), which indicates a specific partial distinction between MBLM and TAU (Table 2).

**Table 2:** Results of the model-based estimated marginal means–pairwise comparisons between the pre-to-post differences of the three groups MBLM, MIN, and TAU.

**Estimated marginal means**								
**Pairwise comparisons**								
**Dependent variable**	**Group**		**Mean difference (I-J)**	**Std. error**	**Sig**.	**95% Confidence interval for difference**		**Effect size Cohen's d**
	**I**	**J**				**Lower CI**	**Upper CI**	
meanNN	MBLM	TAU	60.828	25.653	n.s.	−2.244	123.899	0.75
	MIN	TAU	31.167	27.474	n.s.	−36.383	98.717	0.39
sdNN	MBLM	TAU	4.011	3.438	n.s.	−4.440	12.463	0.43
	MIN	TAU	0.981	3.682	n.s.	−8.070	10.033	0.08
sdaNN5	MBLM	TAU	7.237	4.293	n.s.	−3.316	17.791	0.56
	MIN	TAU	−0.697	4.597	n.s.	−12.000	10.606	−0.05
RMSSD	MBLM	TAU	4.145	1.423	0.015	0.646	7.644	1.05
	MIN	TAU	2.255	1.524	n.s.	−1.493	6.002	0.41
pNN50	MBLM	TAU	0.013	0.006	n.s.	−0.002	0.028	0.65
	MIN	TAU	0.008	0.007	n.s.	−0.008	0.024	0.34
LF/HF	MBLM	TAU	−0.074	1.477	n.s.	−3.706	3.559	−0.01
	MIN	TAU	0.387	1.582	n.s.	−3.504	4.277	0.08
SymDynRenyi4	MBLM	TAU	0.24	0.086	0.021	0.029	0.452	0.86
	MIN	TAU	0.135	0.092	n.s.	−0.091	0.361	0.43

*Based on estimated marginal means. The mean difference is significant at the 0.05 level. Adjustment for multiple comparisons: Sidak*.

Similarly, the MCT resulted in three (out of seven) HRV statistically significant differences between MBLM and TAU, namely for meanNN, *p* = 0.039 (*M*_Diff_ = 60.828, 95%–CI[2.569, 119.086]), RMSSD, *p* = 0.010 (*M*_Diff_ = 4.145, 95%–CI[.913, 7.377]), and SymDynRenyi4, *p* = 0.013 (*M*_Diff_ = 0.240, 95%–CI[.045, 0.435]). Again, the detailed comparison directs to a specific partial distinction of MBLM and TAU (Table 3).

**Table 3:** Results of the observed data in multiple comparison testing between the pre-to-post differences of the three groups MBLM, MIN, and TAU.

**Multiple Comparisons**								
**Dunnett t (2-sided)**								
**Dependent variable**	**Group**		**Mean difference (I-J)**	**Std. error**	**Sig**.	**95% Confidence interval**		**Effect size Cohen's d**
	**I**	**J**				**Lower Bound**	**Upper Bound**	
meanNN	MBLM	TAU	60.828	25.653	0.039	2.569	119.086	0.75
	MIN	TAU	31.167	27.474	n.s.	−31.228	93.562	0.39
sdNN	MBLM	TAU	4.011	3.438	n.s.	−3.795	11.818	0.43
	MIN	TAU	0.981	3.682	n.s.	−7.380	9.342	0.08
sdaNN5	MBLM	TAU	7.237	4.293	n.s.	−2.511	16.986	0.56
	MIN	TAU	−0.697	4.597	n.s.	−11.137	9.744	−0.05
RMSSD	MBLM	TAU	4.145	1.423	0.010	0.913	7.377	1.05
	MIN	TAU	2.255	1.524	n.s.	−1.207	5.716	0.41
pNN50	MBLM	TAU	0.013	0.006	n.s.	−0.001	0.027	0.65
	MIN	TAU	0.008	0.007	n.s.	−0.007	0.023	0.34
LF/HF	MBLM	TAU	−0.074	1.477	n.s.	−3.429	3.282	−0.01
	MIN	TAU	0.387	1.582	n.s.	−3.207	3.980	0.08
SymDynRenyi4	MBLM	TAU	0.240	0.086	0.013	0.045	0.435	0.86
	MIN	TAU	0.135	0.092	n.s.	−0.074	0.344	0.43

*Based on observed means the error term is Mean Square(Error) = 0.081*.

*The mean difference is significant at the 0.05 level. Dunnett t-tests treat one group as a control, and compare all other groups against it*.

No significant differences between MINIMAL and TAU were found in both *post-hoc* analyses.

Effect sizes found in the aforementioned, statistically significant pairwise comparisons *via* EMMEANS and MCT supported the relevance of differences between TAU and MBLM with medium to large effects (see last column in Tables 2, 3).

**Figure 2:**
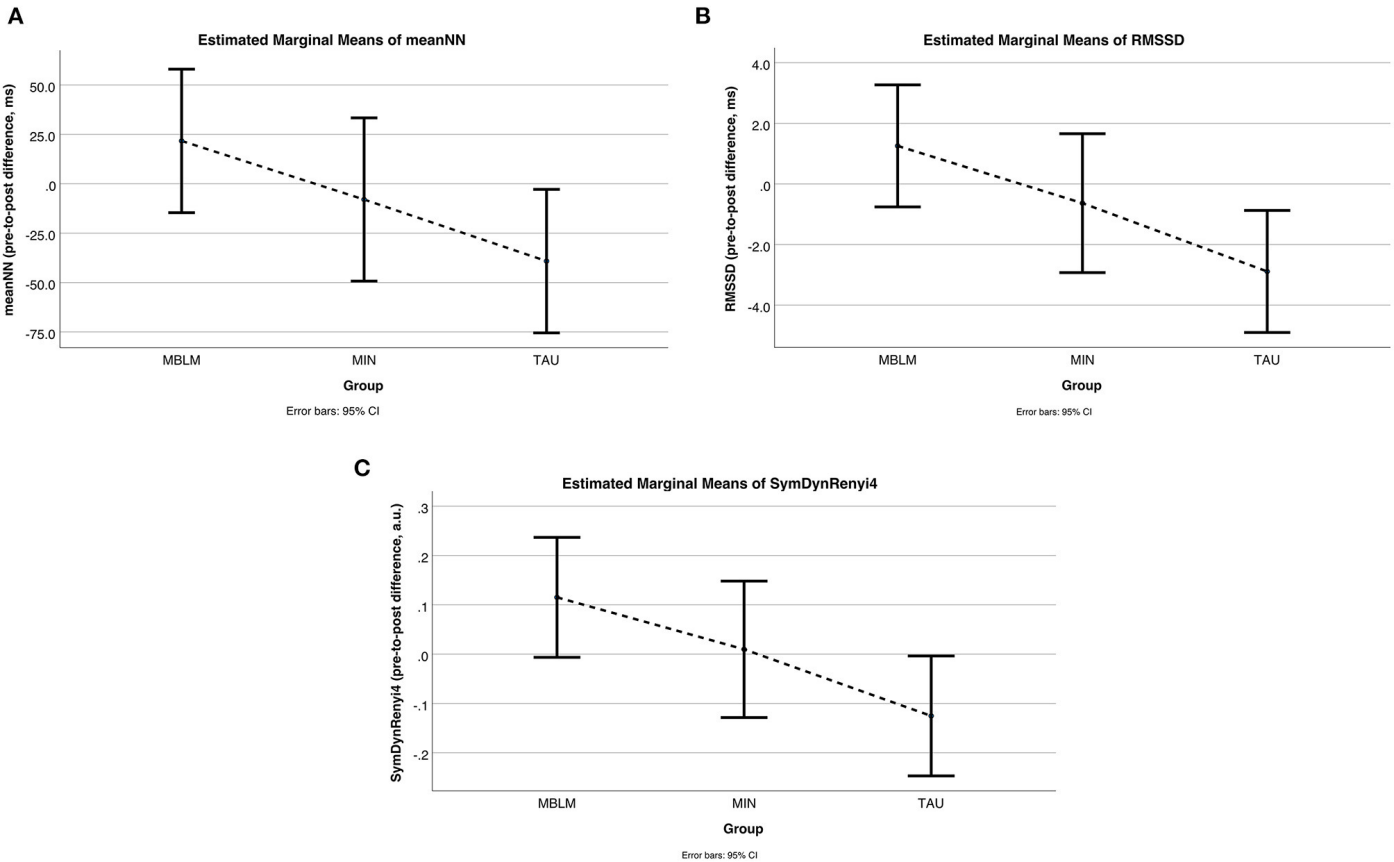
HRV changes (pre-to-post differences) within the three treatment groups. Plots from results of estimated marginal means with pre-to-post differences per group indicating improved HRV for Meditation Based Lifestyle Modification (MBLM), almost no changes for drug continuation therapy (MINIMAL treatment), and deterioration for treatment as usual (TAU); **(A)** Differences in meanNN (average beat-to-beat interval as negative pendant to average heart rate); **(B)** Differences in RMSSD (root mean square of successive beat-to-beat interval differences as measure for short-term or vagal activity); **(C)** Differences in symdynrenyi4 (Renyi4 entropy of symbolic dynamic patterns in the beat-to-beat interval series as measure for its complexity).

Profile plots of the pairwise comparisons in EMMEANS of these parameters showed similar patterns. In the MINIMAL group, there was almost no change, in the TAU group we found a deterioration of the parameters, and in the MBLM group an improvement of HRV features (see Figure 2).

## Discussion

In this exploratory study, we investigated the effects of the Meditation Based Lifestyle Modification program for depressive outpatients on pre-to-post differences in a range of standard HRV parameters and one symbolic dynamics parameter. Intervention effects were compared to effects of a treatment-as-usual protocol based on best clinical practice. Additionally, a group with minimal treatment served as a second comparison to the reference group.

The multivariate analysis of the pre-to-post changes revealed no statistically significant effect between the three groups. However, *post-hoc* tests showed statistically significant differences in the more detailed pairwise comparisons between MBLM and TAU with predominantly large effect sizes in those parameters. There were no significant differences between the MINIMAL group and TAU. The results of the EMMEANS (based on model-fitting) and the MCT (based on observed data) were very similar and thus support each other. This also applied to the effect sizes affirming the results of the GLM *post-hoc* tests. The visualization of the HRV indices validated these findings as well and indicated that the MINIMAL group is almost stagnant, while HRV parameters in the MBLM group increased and decreased in the TAU group.

In detail, the statistically significant differences in the progress of RMSSD and SymDynRenyi4 give evidence of the improved HRV in MBLM and impaired HRV in TAU. The change in resting meanNN (average time between two heartbeats) was also statistically significant distinct between MBLM and TAU. A higher meanNN converts to a lower beats-per-min index at rest for the MBLM group compared to a faster beating heart in the outcome of TAU participants. This results in less stress on the heart and thus a healthier condition while resting in the MBLM group. Similarly, the directions of change in the non-significant parameters (sdNN, sdaNN5 and pNN50) showed the same trend. The only exception was the almost constant LF/HF parameter in all three groups. However, recent studies demonstrated that sympatho-vagal balance may not be accurately measured *via* the LF/HF ratio (44).

In terms of HRV, the RMSSD reflects the parasympathetic activity and therefore the adaptation capacity of the autonomic regulation. More specifically, the RMSSD is a measure for the short-term variations in the heart rate series and correlates to the high frequency (HF, 0.15-0.4 Hz) component in frequency domain (9). As mentioned, the parasympathetic (vagal) activity is the primary driver of this component and acts as antagonist to the sympathetic activity. An imbalance of these two parts of the autonomic nervous system or a predominant sympathetic activity (especially at resting condition) is seen in relation to several physiological and psychological diseases (9, 45). Therefore, an increased RMSSD attests an improved HRV and a potential benefit to personal health for the participants of MBLM compared to the ones of the TAU group. Regarding the Rényi entropy (with α = 4) of the occurring symbolic patterns in the NN series (SymDynRenyi4), higher values admit more diversity and thus enhanced HRV for MBLM against the impaired HRV for TAU. Again, this leads to a potential advantage in overall health for MBLM participants.

While the improvements for the participants in the intervention group and the almost unchanged parameters for participants in the minimal treatment group was in line with our expectations, the deterioration of HRV parameters found in the multimodal treatment group were unexpected. One of the reasons for this could be that the standard treatment caused more stress to patients in general than the MBLM program, which is based on positive psychology and aimed at achieving greater harmonization. In a qualitative analysis of the MBLM program compared to standard treatment, evidence was found that the MBLM program was subjectively experienced as more coherent and more tailored to individual needs than the standard therapy (33), indicating a source of stress that might have influenced HRV parameters–at least during the first 8 weeks of therapy.

In general, there is broad agreement that MBIs using techniques such as mindfulness, yoga, or meditation contribute to better health and treatment outcomes for depression (46–48). Taking HRV into account, a direct comparison to other studies and literature is quite difficult as we present results of a new approach in treatment of depressive disorders with the novel MBLM program which combines mind-body techniques like yoga and meditation with (positive) psychotherapy. Additionally, our study focused on the treatment comparison of three groups based on their pre-to-post differences in HRV indices. We therefore did not investigate HRV changes over time within the individual groups. In other words, our statistical analysis was set up to consider group effects rather than time effects. When considering the sign and absolute value of our HRV differences, we may estimate the direction of changes and draw a loose comparison. That way, we see some similarities to a study by Shapiro et al. (49), which reports a significant reduction in LF component after a yoga intervention program for depressive patients while HF and the LF/HF ratio remained unchanged. In our study, we could confirm the results for unaffected LF/HF in all three groups (data not shown). Concerning the unchanged HF, which is a correlative parameter to RMSSD as both reflect the vagal activity (in parasympathetic nervous system), our results differ somewhat. Due to increases in the MBLM group and decreases in the TAU group, the RMSSD difference in our comparison is significant. These pre-post changes are shown in Figure 2. A systematic review on the effects of MBIs on HRV in different populations by Zou et al. (1) stated significantly decreased normalized LF (LFn) and increased normalized HF (HFn) as well as reduced LF/HF ratio due to Tai Chi or yoga interventions. Concerning the LF/HF findings, they found no significant difference in the effect of the MBI between healthy and clinical populations (including depressive symptoms). Furthermore, a yoga-meditation intervention for depressive women resulted in increased HF and decreased LF as well as LF/HF HRV indices compared to no changes in a control group (50). These findings may support those of Zou et al. (1), and we can at least confirm the improved vagal activity (reflected by HF-HRV) with the increased RMSSD values in our study. But our results do not support the finding of decreased LF/HR balance due to our MBLM treatment program. On the other hand a study evaluating a cognitive behavioral therapy program combining mindfulness and yoga methods on posttraumatic stress disorder symptoms (including depression) found no pre-post changes in vagal activity reflected by HF-HRV or RMSSD (51). Again, in contrast, our results instead tend to suggest an improvement in vagally mediated RMSSD. However, in all of these comparisons, it should be noted that subjects and treatments differ. Moreover, the mentioned studies show an inconsistency in their findings on frequency HRV parameters and a lack of additional time domain or non-linear HRV indices. Additionally, we examined the treatment effect on HRV only for specific subjects with mild to moderate depression and not for major depression patients or healthy populations. Furthermore, the reported studies in the meta-analysis utilized HRV measurements between 5 and 20 min, which could make a difference for comparison. Studies did report on filtering methods and adjustment for ectopic beats or artifacts, but not on the condition of stationarity of the investigated tachograms. Many HRV indices, especially frequency domain analysis based on Fast Fourier Transformation is known to be sensitive to artifact distortions and non–stationarities (9, 41). Due to a controlled randomization process, we exclude gender dependencies in our data, which may be biased in patients with depressive disorder (52).

A joint evaluation of clinical parameters with HRV parameters has not yet been performed. The clinical part of this evaluation study was evaluated and published separately. Nevertheless, as a small excerpt from the clinical evaluation, we can provide the changes in BDI-II scores for cross-comparison and brief classification of our study results here (see Table 4). Based on these outcomes, a considerable benefit was found for the MBLM group compared to the other two groups.

**Table 4:** Changes in BDI-II scores from clinical evaluation.

**Outcome**	**Group**	**Baseline**	**8 weeks**	***ΔM***
BDI-II	MINIMAL	23.30 (8.40)	21.59 (9.67)	−1.71
	TAU	26.04 (8.12)	22.70 (9.26)	−3.34
	MBLM	26.74 (9.46)	13.59 (10.63)	−13.15

*BDI-II, Beck Depression Inventory; 2. Revision 1996 (36)*.

This study is not without limitations. Although randomized and stratified regarding certain clinical and sociodemographic variables, the groups may have differed in other aspects relevant to HRV outcomes, like regular physical activity. Furthermore, we conducted a short-term HRV analysis within a time window of 20-min out of 24-h of recording. Long-term HRV analysis would probably result in additional findings but would also bring further problems in signal processing (e.g., more distortions and artifacts, search for appropriate window of stationarity). Also, short-term recordings based on mobile 24-h recordings allowed less control about the measurement conditions for the investigator (e.g., concerning physical condition, mood). We tried to address this by searching for a window of stationarity, with good signal quality and individually typical heart rate progression avoiding periods of very high or low load. In addition, this study should be considered exploratory, with analyses intended to provide a first impression and provide a basis for further investigation.

For future research, we suggest replicating our basic findings in study designs with higher statistical power. In addition, HRV parameters during night phases should also be considered separately. Further approaches would be long-term analyses (e.g., over 24 h) and foci on dynamic processes in the derived signals. This could provide even more insights on the sympatho-vagal balance. Also, it might be useful to acquire more physiological data additionally to the ECG. For instance, measuring (continuous) blood pressure or the easy to obtain pulse wave from photoplethysmography (*via* pulse oximeter) would provide information about cardiovascular processes by analyzing the blood pressure variability (BPV) or pulse wave variability (PWV). An example for utilizing the PWV in evaluating an MBI (based on MBSR) was given by Voss, Bogdanski (53). The same study also highlights, that cardiovascular functions in particular are affected by this MBI Training. In addition, another study supports the potential association between depression and changes in cardiovascular function measured *via* HRV and BPV (54). Furthermore, the response in blood pressure during meditation seems to differ from other relaxing activities and suggests a link between the intervention and the ANS (55). Sung, Woo (56) also assume a connection between alterations in BPV and quality of life indices. Finally, Parati, Ochoa (57) reviewed blood pressure and the potential of its variability indices as predictors in clinical applications. This may also cover the risk evaluation of cardiovascular morbidity and mortality due to insufficient stress management or coping and possibly highlight the benefit of MBIs in treatment of mental disorders like depression. At least, the additional physiological information would offer the opportunity of assessing bivariate parameters representing the coupling of HRV and BPV/PWV. Finally, a joint analysis of clinical (such as BDI score) and HRV parameters could be of interest.

As a result of this exploratory HRV analysis, we can conclude that the MBLM program seems to strengthen some functions of the autonomic regulation and mediates a better stress adaption. Based on our findings and the supportive literature about the theoretical and clinical effects of MBIs, a psychophysiological effect can be assumed. Our study points to a health benefit for outpatients with mild to moderate depression after participating in the MBLM program compared to treatment as usual. Further methods and indices from different domains of biosignal analysis (like non-linear dynamics) could provide additional insights.

## Data Availability Statement

The raw data supporting the conclusions of this article will be made available by the authors, upon reasonable request.

## Ethics Statement

The studies involving human participants were reviewed and approved by Chemnitz University of Technology Protocol # V-276-15-PS-MBLM-D- 14062018. The patients/participants provided their written informed consent to participate in this study.

## Author Contributions

HB conceptualized and designed the study. AV, HB, GS, and MB contributed to statistical analysis and reporting. HB and MB wrote the first draft of the manuscript. All authors worked on the final version of the manuscript.

## Funding

This project was funded by Karl and Veronica Carstens Foundation under Award Numbers KVC 0/098/2018.

## Conflict of Interest

The authors declare that the research was conducted in the absence of any commercial or financial relationships that could be construed as a potential conflict of interest.

## Publisher's Note

All claims expressed in this article are solely those of the authors and do not necessarily represent those of their affiliated organizations, or those of the publisher, the editors and the reviewers. Any product that may be evaluated in this article, or claim that may be made by its manufacturer, is not guaranteed or endorsed by the publisher.

## References

[1] Zou L Sasaki JE Wei GX Huang T Yeung AS Neto OB. Effects of mind(-)body exercises (tai chi/yoga) on heart rate variability parameters and perceived stress: a systematic review with meta-analysis of randomized controlled trials. J Clin Med. (2018) 7:404. 10.3390/jcm7110404 30384420 PMC6262541

[2] Pascoe MC Thompson DR Ski CF. Yoga, mindfulness-based stress reduction and stress-related physiological measures: a meta-analysis. Psychoneuroendocrinology. (2017) 86:152–68. 10.1016/j.psyneuen.2017.08.008 28963884

[3] Tyagi A Cohen M. Yoga and heart rate variability: a comprehensive review of the literature. Int J Yoga. (2016) 9:97–113. 10.4103/0973-6131.183712 27512317 PMC4959333

[4] van der Zwan JE de Vente W Huizink AC Bogels SM de Bruin EI. Physical activity, mindfulness meditation, or heart rate variability biofeedback for stress reduction: a randomized controlled trial. Appl Psychophysiol Biofeedback. (2015) 40:257–68. 10.1007/s10484-015-9293-x 26111942 PMC4648965

[5] Telles S Singh D Naveen KV Pailoor S Singh N Pathak S. P300 and heart rate variability recorded simultaneously in meditation. Clin EEG Neurosci. (2019) 50:161–71. 10.1177/1550059418790717 30056746

[6] Arya NK Singh K Malik A Mehrotra R. Effect of heartfulness cleaning and meditation on heart rate variability. Indian Heart J. (2018) 70:S50–S5. 10.1016/j.ihj.2018.05.004 30595318 PMC6309138

[7] Kirk U Axelsen JL. Heart rate variability is enhanced during mindfulness practice: a randomized controlled trial involving a 10-day online-based mindfulness intervention. PLoS ONE. (2020) 15:e0243488. 10.1371/journal.pone.0243488 33332403 PMC7746169

[8] Brown L Rando AA Eichel K Van Dam NT Celano CM Huffman JC. The effects of mindfulness and meditation on vagally mediated heart rate variability: a meta-analysis. Psychosom Med. (2021) 83:631–40. 10.1097/PSY.0000000000000900 33395216 PMC8243562

[9] TaskForce. Heart rate variability: standards of measurement, physiological interpretation and clinical use. Task Force of the european society of cardiology and the north american society of pacing and electrophysiology. Circulation. (1996) 1:1043–65. 8598068

[10] Kleiger RE Stein PK Bigger JT. Jr. Heart rate variability: measurement and clinical utility. Ann Noninvasive Electrocardiol. (2005) 10:88–101. 10.1111/j.1542-474X.2005.10101.x 15649244 PMC6932537

[11] Malik M. Heart rate variability. Curr Opin Cardiol. (1998) 13:36–44. 10.1097/00001573-199801000-00006 9559255

[12] Gang Y Malik M. Heart rate variability analysis in general medicine. Indian Pacing Electrophysiol J. (2003) 3:34–40. 16943988 PMC1555630

[13] Ghiadoni L Donald AE Cropley M Mullen MJ Oakley G Taylor M. Mental stress induces transient endothelial dysfunction in humans. Circulation. (2000) 102:2473–8. 10.1161/01.CIR.102.20.2473 11076819

[14] Strike P. Systematic review of mental stress-induced myocardial ischaemia. Eur Heart J. (2003) 24:690–703. 10.1016/S0195-668X(02)00615-2 12713764

[15] Chandola T Britton A Brunner E Hemingway H Malik M Kumari M. Work stress and coronary heart disease: what are the mechanisms? Eur Heart J. (2008) 29:640–8. 10.1093/eurheartj/ehm584 18216031

[16] Schwartz BG French WJ Mayeda GS Burstein S Economides C Bhandari AK. Emotional stressors trigger cardiovascular events. Int J Clin Pract. (2012) 66:631–9. 10.1111/j.1742-1241.2012.02920.x 22698415

[17] Wirtz PH von Kanel R. Psychological stress, inflammation, and coronary heart disease. Curr Cardiol Rep. (2017) 19:111. 10.1007/s11886-017-0919-x 28932967

[18] Voss A Hnatkova K Wessel N Kurths J Sander A Schirdewan A. Multiparametric analysis of heart rate variability used for risk stratification among survivors of acute myocardial infarction. Pacing Clin Electrophysiol. (1998) 21:186–92. 10.1111/j.1540-8159.1998.tb01086.x 9474670

[19] Voss A Fischer C Schroeder R Figulla HR Goernig M. Segmented poincare plot analysis for risk stratification in patients with dilated cardiomyopathy. Methods Inf Med. (2010) 49:511–5. 10.3414/ME09-02-0050 20526525

[20] Voss A Kurths J Kleiner HJ Witt A Wessel N Saparin P. The application of methods of non-linear dynamics for the improved and predictive recognition of patients threatened by sudden cardiac death. Cardiovasc Res. (1996) 31:419–33. 10.1016/S0008-6363(96)00008-9 8681329

[21] Ader R. Psychoneuroimmunology. Curr Dir Psychol Sci. (2001) 10:94–8. 10.1111/1467-8721.00124

[22] Glaser R Kiecolt-Glaser J. How stress damages immune system and health. Discov Med. (2005) 5:165–9. 10.1038/nri1571 20704904

[23] Tian R Hou G Li D Yuan T-F. A possible change process of inflammatory cytokines in the prolonged chronic stress and its ultimate implications for health. Scientific World Journal. (2014) 2014:8. 10.1155/2014/780616 24995360 PMC4065693

[24] Connor TJ Leonard BE. Depression, stress and immunological activation: the role of cytokines in depressive disorders. Life Sci. (1998) 62:583–606. 10.1016/S0024-3205(97)00990-9 9472719

[25] Marin MF Lord C Andrews J Juster RP Sindi S Arsenault-Lapierre G. Chronic stress, cognitive functioning and mental health. Neurobiol Learn Mem. (2011) 96:583–95. 10.1016/j.nlm.2011.02.016 21376129

[26] Koschke M Boettger MK Schulz S Berger S Terhaar J Voss A. Autonomy of autonomic dysfunction in major depression. Psychosom Med. (2009) 71:852–60. 10.1097/PSY.0b013e3181b8bb7a 19779146

[27] Shinba T Murotsu K Usui Y Andow Y Terada H Takahashi M. Usefulness of heart rate variability indices in assessing the risk of an unsuccessful return to work after sick leave in depressed patients. Neuropsychopharmacol Rep. (2020) 40:239–45. 10.1002/npr2.12121 32627417 PMC7722666

[28] Kircanski K Williams LM Gotlib IH. Heart rate variability as a biomarker of anxious depression response to antidepressant medication. Depress Anxiety. (2019) 36:63–71. 10.1002/da.22843 30311742 PMC6318007

[29] Lin IM Fan SY Yen CF Yeh YC Tang TC Huang MF. Heart rate variability biofeedback increased autonomic activation and improved symptoms of depression and insomnia among patients with major depression disorder. Clin Psychopharmacol Neurosci. (2019) 17:222–32. 10.9758/cpn.2019.17.2.222 30905122 PMC6478078

[30] Taylor AG Goehler LE Galper DI Innes KE Bourguignon C. Top-down and bottom-up mechanisms in mind-body medicine: development of an integrative framework for psychophysiological research. Explore (NY). (2010) 6:29–41. 10.1016/j.explore.2009.10.004 20129310 PMC2818254

[31] Gard T Noggle JJ Park CL Vago DR Wilson A. Potential self-regulatory mechanisms of yoga for psychological health. Front Hum Neurosci. (2014) 8:770. 10.3389/fnhum.2014.00770 25368562 PMC4179745

[32] Bringmann HC Bringmann N Jeitler M Brunnhuber S Michalsen A Sedlmeier P. Meditation-based lifestyle modification: development of an integrative mind-body program for mental health and human flourishing. Complement Med Res. (2021) 28:252–62. 10.1159/000512333 33285545

[33] Bringmann HC Vennemann J Gross J Matko K Sedlmeier P. “To be finally at peace with myself”: a qualitative study reflecting experiences of the meditation-based lifestyle modification program in mild-to-moderate depression. J Altern Complement Med. (2021) 27:786–95. 10.1089/acm.2021.0038 34185550

[34] Bringmann HC Bringmann N Jeitler M Brunnhuber S Michalsen A Sedlmeier P. Meditation Based Lifestyle Modification (MBLM) in outpatients with mild to moderate depression: a mixed-methods feasibility study. Complement Ther Med. (2021) 56:102598. 10.1016/j.ctim.2020.102598 33212169

[35] Matko K Sedlmeier P Bringmann HC. Differential effects of ethical education, physical hatha yoga, and mantra meditation on well-being and stress in healthy participants-an experimental single-case study. Front Psychol. (2021) 12:672301. 10.3389/fpsyg.2021.672301 34421729 PMC8375679

[36] Beck AT Steer RA Ball R Ranieri W. Comparison of beck depression inventories -IA and -II in psychiatric outpatients. J Pers Assess. (1996) 67:588–97. 10.1207/s15327752jpa6703_13 8991972

[37] O'Callaghan CA. OxMaR: open source free software for online minimization and randomization for clinical trials. PLoS ONE. (2014) 9:e110761. 10.1371/journal.pone.0110761 25353169 PMC4213009

[38] Scott NW McPherson GC Ramsay CR Campbell MK. The method of minimization for allocation to clinical trials. a review Control Clin Trials. (2002) 23:662–74. 10.1016/S0197-2456(02)00242-8 12505244

[39] für die Leitliniengruppe Unipolare Depression*. S3-Leitlinie/Nationale VersorgungsLeitlinie Unipolare Depression – Kurzfassung, 2. Auflflage. Version 1. DGPPN, BÄK, KBV, AWMF (2017). 10.6101/AZQ/000366

[40] The MathWorks I. R Wave Detection in the ECG. The MathWorks, Inc. (2021). Available online at: https://de.mathworks.com/help/wavelet/ug/r-wave-detection-in-the-ecg.html (accessed November 3, 2021).

[41] Magagnin V Bassani T Bari V Turiel M Maestri R Pinna GD. Non-stationarities significantly distort short-term spectral, symbolic and entropy heart rate variability indices. Physiol Meas. (2011) 32:1775–86. 10.1088/0967-3334/32/11/S05 22027399

[42] Kurths J Voss A Saparin P Witt A Kleiner HJ Wessel N. Quantitative analysis of heart rate variability. Chaos. (1995) 5:88–94. 10.1063/1.166090 12780160

[43] Cohen J. Statistical Power Analysis for the Behavioral Sciences: L. Erlbaum Associates. New York, NY: Lawrence Erlbaum Associates (1988).

[44] von Rosenberg W Chanwimalueang T Adjei T Jaffer U Goverdovsky V Mandic DP. Resolving ambiguities in the LF/HF Ratio: LF-HF scatter plots for the categorization of mental and physical stress from HRV. Front Physiol. (2017) 8:360. 10.3389/fphys.2017.00360 28659811 PMC5469891

[45] Rajendra Acharya U Paul Joseph K Kannathal N Lim CM Suri JS. Heart rate variability: a review. Med Biol Eng Comput. (2006) 44:1031–51. 10.1007/s11517-006-0119-0 17111118

[46] Hofmann SG Gomez AF. Mindfulness-based interventions for anxiety and depression. Psychiatr Clin North Am. (2017) 40:739–49. 10.1016/j.psc.2017.08.008 29080597 PMC5679245

[47] Wielgosz J Goldberg SB Kral TRA Dunne JD Davidson RJ. Mindfulness meditation and psychopathology. Annu Rev Clin Psychol. (2019) 15:285–316. 10.1146/annurev-clinpsy-021815-093423 30525995 PMC6597263

[48] Saeed SA Cunningham K Bloch RM. Depression and anxiety disorders: benefits of exercise, yoga, and meditation. Am Fam Physician. (2019) 99:620–7. 31083878

[49] Shapiro D Cook IA Davydov DM Ottaviani C Leuchter AF Abrams M. Yoga as a complementary treatment of depression: effects of traits and moods on treatment outcome. Evid Based Complement Alternat Med. (2007) 4:493–502. 10.1093/ecam/nel114 18227917 PMC2176141

[50] Chu IH Wu WL Lin IM Chang YK Lin YJ Yang PC. Effects of yoga on heart rate variability and depressive symptoms in women: a randomized controlled trial. J Altern Complement Med. (2017) 23:310–6. 10.1089/acm.2016.0135 28051319

[51] Kirk MA Taha B McCague H Dang K Hatzinakos D Katz J. An online cognitive behavioral therapy, mindfulness meditation, and yoga (CBT-MY) intervention for posttraumatic stress disorder: psychometric and psychophysiology outcomes. JMIR Ment Health. (2021). 10.2196/preprints.26479 PMC8922150 34499613

[52] Voss A Boettger MK Schulz S Gross K Bar KJ. Gender-dependent impact of major depression on autonomic cardiovascular modulation. Prog Neuropsychopharmacol Biol Psychiatry. (2011) 35:1131–8. 10.1016/j.pnpbp.2011.03.015 21453741

[53] Voss A Bogdanski M Langohr B Albrecht R Sandbothe M. Mindfulness-based student training leads to a reduction in physiological evaluated stress. Front Psychol. (2020) 11:645. 10.3389/fpsyg.2020.00645 32477199 PMC7240125

[54] Schulz S Koschke M Bar KJ Voss A. The altered complexity of cardiovascular regulation in depressed patients. Physiol Meas. (2010) 31:303–21. 10.1088/0967-3334/31/3/003 20086275

[55] Ditto B Eclache M Goldman N. Short-term autonomic and cardiovascular effects of mindfulness body scan meditation. Ann Behav Med. (2006) 32:227–34. 10.1207/s15324796abm3203_9 17107296

[56] Sung J Woo JM Kim W Lim SK Chung AS. Relationship between blood pressure variability and the quality of life. Yonsei Med J. (2014) 55:374–8. 10.3349/ymj.2014.55.2.374 24532506 PMC3936645

[57] Parati G Ochoa JE Lombardi C Bilo G. Blood pressure variability: assessment, predictive value, and potential as a therapeutic target. Curr Hypertens Rep. (2015) 17:537. 10.1007/s11906-015-0537-1 25790801

