# Supplementary material for: Impact of Meditation–Based Lifestyle Modification on HRV in Outpatients With Mild to Moderate Depression: An Exploratory Study
Source: Front Psychiatry. 2022 Jun 9;13:808442. doi: 10.3389/fpsyt.2022.808442 (PMC9218213; doi:10.3389/fpsyt.2022.808442)
Supplement: Supplementary file 1 [file Table_1.DOCX]

# Supplementary Materials

SPSS Syntax for performing MANOVA using the General Linear Model (GLM) function with post-hoc testing and multiple comparisons

GLM

meanNN

sdNN

sdaNN5

rmssd

pNN50

LFHF

SymDynRenyi4

BY Group

/CONTRAST(Group)=SIMPLE(3)

/METHOD=SSTYPE(3)

/INTERCEPT=INCLUDE

/POSTHOC=Group(DUNNETT)

/PLOT=PROFILE(Group) TYPE=LINE ERRORBAR=CI MEANREFERENCE=NO YAXIS=AUTO

/EMMEANS=TABLES(Group) COMPARE ADJ(SIDAK)

/PRINT=DESCRIPTIVE ETASQ HOMOGENEITY

/CRITERIA=ALPHA(.05)

/DESIGN= Group.
