# Supplementary material for: Impact of Meditation–Based Lifestyle Modification on HRV in Outpatients With Mild to Moderate Depression: An Exploratory Study
Source: Front Psychiatry. 2022 Jun 9;13:808442. doi: 10.3389/fpsyt.2022.808442 (PMC9218213; doi:10.3389/fpsyt.2022.808442)
Supplement: Supplementary file 2 [file Table_2.DOCX]

Supplementary Material

# Supplementary Figures and Tables

## Supplementary Figures

**Supplementary Figure S1.** Concept of novel Meditation Based Lifestyle Modification (MBLM) mind-body program for treatment of mild-to-moderate depressive outpatients.
